# Limb girdle muscular dystrophy type 2G with myopathic-neurogenic motor unit potentials and a novel muscle image pattern

**DOI:** 10.1186/1472-6890-14-41

**Published:** 2014-10-04

**Authors:** Ana Cotta, Julia Filardi Paim, Antonio Lopes da-Cunha-Junior, Rafael Xavier Neto, Simone Vilela Nunes, Monica Magalhaes Navarro, Jaquelin Valicek, Elmano Carvalho, Lydia U Yamamoto, Camila F Almeida, Shelida Vasconcelos Braz, Reinaldo Issao Takata, Mariz Vainzof

**Affiliations:** 1 Department of Pathology, SARAH Network of Rehabilitation Hospitals, Belo Horizonte, Brazil; 2 Department of Radiology, SARAH Network of Rehabilitation Hospitals, Belo Horizonte, Brazil; 3 Department of Neurology, SARAH Network of Rehabilitation Hospitals, Belo Horizonte, Brazil; 4 Departments of Pediatrics and Genetics, SARAH Network of Rehabilitation Hospitals, Belo Horizonte, Brazil; 5 Department of Neurophysiology, SARAH Network of Rehabilitation Hospitals, Belo Horizonte, Brazil; 6 Human Genome Research Center, University of São Paulo, São Paulo, Brazil; 7 Department of Electron Microscopy, SARAH Network of Rehabilitation Hospitals, Brasília, Brazil; 8 Department of Molecular Biology, SARAH Network of Rehabilitation Hospitals, Brasília, Brazil

**Keywords:** Limb girdle muscular dystrophy, Telethonin, Neurogenic, Computed tomography, Rimmed vacuoles

## Abstract

**Background:**

Limb girdle muscular dystrophy type 2G (LGMD2G) is a subtype of autosomal recessive muscular dystrophy caused by mutations in the telethonin gene. There are few LGMD2G patients worldwide reported, and this is the first description associated with early tibialis anterior sparing on muscle image and myopathic-neurogenic motor unit potentials.

**Case presentation:**

Here we report a 31 years old caucasian male patient with progressive gait disturbance, and severe lower limb proximal weakness since the age of 20 years, associated with subtle facial muscle weakness. Computed tomography demonstrated soleus, medial gastrocnemius, and diffuse thigh muscles involvement with tibialis anterior sparing. Electromyography disclosed both neurogenic and myopathic motor unit potentials. Muscle biopsy demonstrated large groups of atrophic and hypertrophic fibers, frequent fibers with intracytoplasmic rimmed vacuoles full of autophagic membrane and sarcoplasmic debris, and a total deficiency of telethonin. Molecular investigation identified the common homozygous c.157C > T in the *TCAP* gene.

**Conclusion:**

This report expands the phenotypic variability of telethoninopathy/ LGMD2G, including: 1) mixed neurogenic and myopathic motor unit potentials, 2) facial weakness, and 3) tibialis anterior sparing. Appropriate diagnosis in these cases is important for genetic counseling and prognosis.

## Background

Limb girdle muscular dystrophy 2G (LGMD2G) is a subtype of autosomal recessive muscular dystrophy caused by mutations in the telethonin (*TCAP*) gene, characterized by a progressive limb girdle muscular weakness. Its progression rate has been considered relatively slow, compared to other muscular dystrophies [1]. There is a worldwide small number of known patients with telethoninopathy, either with limb girdle muscular dystrophy 2G [2-9] or with congenital muscular dystrophy [1,10,11]. The number of publications with image studies in LGMD2G patients is even smaller [7,8,12].

The aim of this study is to present clinical, neurophysiologic, laboratorial, muscle morphologic, immunohistochemical and electron microscopy analyses and molecular findings in a newly identified patient with LGMD2G, revealing a higher phenotypic variability. This is the first report of a telethoninopathy patient with early tibialis anterior muscular sparing on image study, facial weakness, myopathic/neurogenic motor unit potentials, and ultrastructural study of the rimmed vacuoles on muscle biopsy [2-12].

## Case presentation

### Patient information

A 29 years old male patient was admitted at the outpatient clinic. He reported frequent falls since gait acquisition when he was one year old. After 20 years old, he noticed progressive gait disturbance with weakness in the lower limbs and tremor on the hands. No history of parental consanguinity or similar cases in the family were reported. Physical examination at admission revealed increased calf, difficulties performing extraocular eye movement and lower limb proximal weakness.

### Clinical findings

Clinical examination, included muscle strength testing as rated by the Medical Research Council scale. The degree of muscle involvement on image studies was evaluated according to a 5-point scale [13,14]. Stage 0 is normal appearance; stage 1 (mild) corresponds to traces of decreased density on CT (computed tomography) or increased signal intensity on the T1-weighted MR (magnetic resonance) sequences; stage 2 (moderate) reflects reduced density on CT or increased signal intensity (MRI) with beginning confluence in less than 50% of the muscle; stage 3 (severe) refers to decreased density (CT) or increased signal intensity (MRI) in more than 50% of an examined muscle; and stage 4 (advanced disease) to a state when the entire muscle is replaced by lower density (CT) or increased signal intensity (MRI).

### Diagnostic assessment

Electromyography (EMG) disclosed a mixed pattern, neurogenic and myopathic, mainly myopathic, with mild muscle membrane hyperexcitability. We analyzed the right deltoideus, triceps, biceps brachialis and first dorsal interosseous, left vastus lateralis, bilateral tibialis anterior and right gastrocnemius medialis by conventional and quantitative EMG method with twenty motor unit potentials (MUP) quantification of each muscle studied. At rest, fibrillation and positive waves were detected in the right gastrocnemius medialis. At initial effort, all muscles studied showed low amplitude and/or short duration MUP (myopathic characteristics), mainly in the lower limbs. Right triceps brachialis, deltoideus and first dorsal interosseous muscles presented a mixed pattern (myopathic and neurogenic), showing besides myopathic motor unit potentials, many other potentials with high amplitude and/or long duration (neurogenic characteristics), with high MUP polyphasic proportion in all of them (Table 1).

**Table 1:** Quantitative analyses of motor unit potentials in the muscles studied

**Muscle N = 20 MUP per muscle**	**Amplitude (μV) Mean (min-max)**	**Duration (ms) Mean (min-max)**	**Polyphase (%)**
Right deltoideus	4322 (1221–10000)	6,1 (3,6-10,4)	45
Right triceps	5243 (679–8611)	10,1 (3,6-17,4)	30
Right biceps brachialis	1608 (774–3953)	6,3 (2,8-12,2)	35
Right first dorsal interosseous	5265 (1399–10000)	7,1 (1,8-13,6)	25
Left vastus lateralis	1329 (454–4233)	5,9 (3,4-9,0)	20
Right tibialis anterior	1619 (292–3047)	6,2 (3,4-9,0)	30
Left tibialis anterior	1279 (413–2472)	4,8 (2,2-10,6)	40
Right gastrocnemius medialis	NP	NP	NP

MUP- motor unit potentials; NP- Not performed.

**Figure 1:**
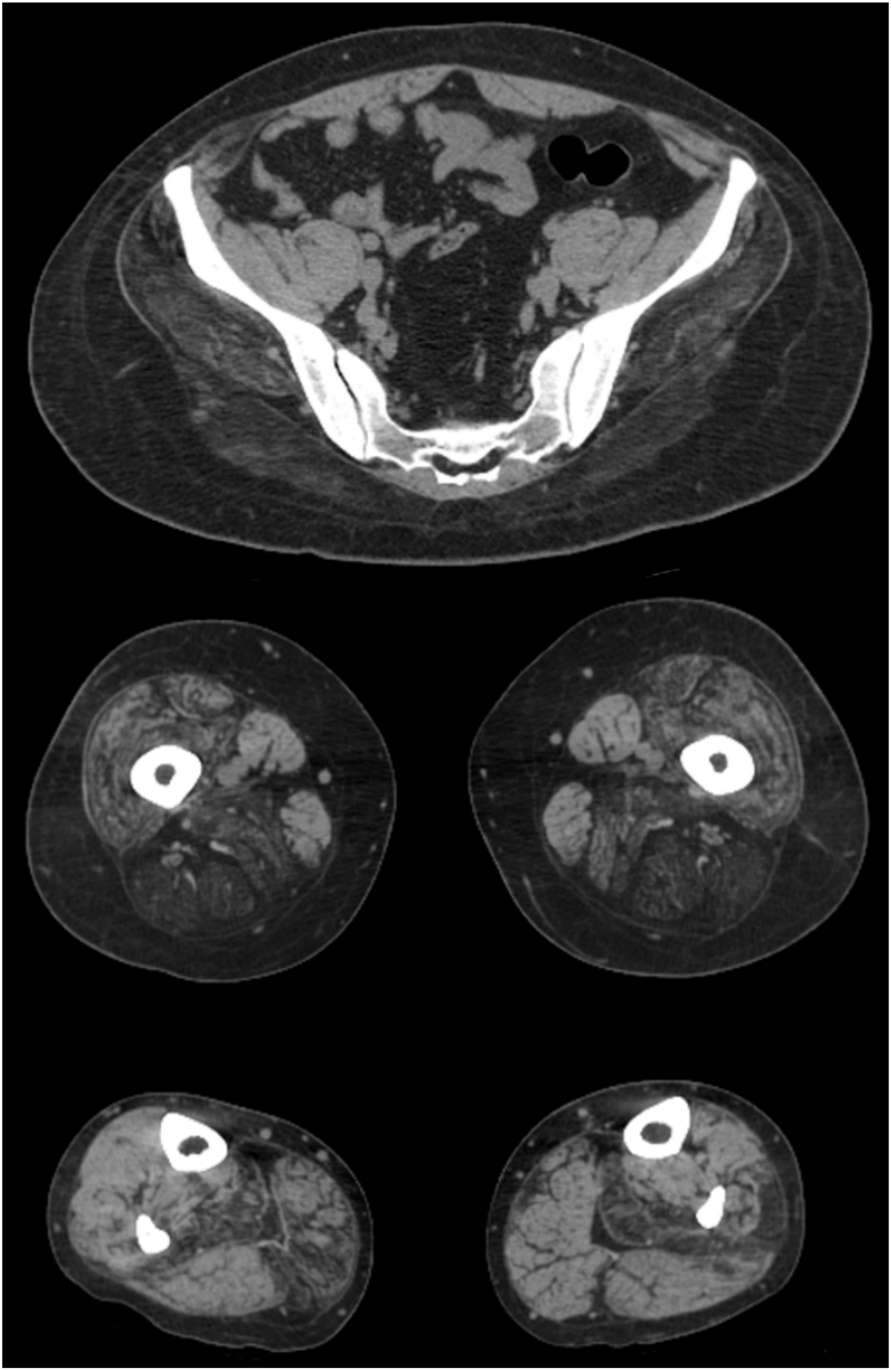
**Computed tomography of the pelvis, thighs and legs.** At the pelvis there is advanced gluteus maximus, gluteus medius, and gluteus minimus involvement. At the thigh, there is advanced biceps femoris, semitendinosus, semimembranosus involvement; severe vastus lateralis, vastus medialis, vastus intermedius, and adductor magnus involvement, with relative preservation and hypertrophy of sartorius and gracilis. At the leg, there is asymmetric muscle involvement with advanced right gastrocnemius medialis, left peroneus, bilateral soleus involvement, compared to tibialis anterior muscle sparing.

**Figure 2:**
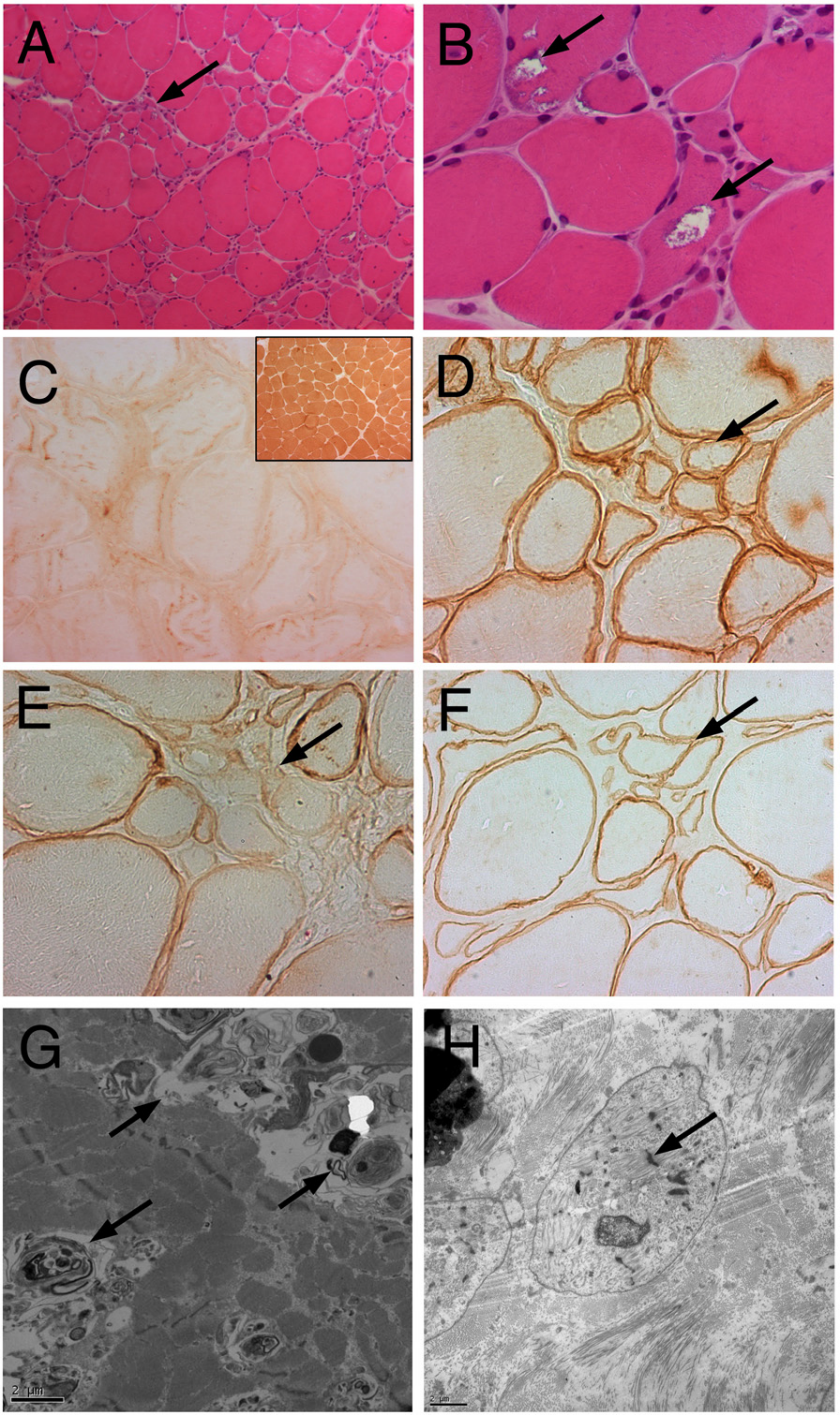
**Muscle biopsy. (A)** Severe variation in fiber calibre, showing groups of atrophic fibers (arrow) and groups of hypertrofic fibers (HE 100x). **(B)** Frequent rimmed vacuoles (arrows) with basophilic material (HE 400×). **(C)** Immunohistochemical reaction with complete telethonin deficiency. Inset presents strong normal sarcolemmal reaction in control muscle (Antibody anti-telethonin (G-11): sc-25327, Santa Cruz Biotechnology, Inc., at 1:50 dilution). **(D, E, F)** Serial frozen sections of the same muscle fibers (arrows) showing irregular dystrophin carboxy-terminus reaction **(E)** (D - Rod-domain anti-dystrophin antibody (NCLDYS1 Novocastra at 1:20 dilution), E - Carboxy-terminus anti-dystrophin antibody (NCLDYS2 Novocastra at 1:20 dilution), F - Amino-terminus anti-dystrophin antibody (NCLDYS3 Novocastra at 1:20 dilution), **(G)** Muscle tissue shows autophagic vacuoles (arrows) with membrane debris and myelinoid figures (transmission electron microscopy 5000x), **(H)** Atrophic muscle fiber with myofibrillar disorganization, and few residual sarcomeres with distinguishable Z lines (arrows) (transmission electron microscopy 6000x).

Serum muscle enzymes were increased with a total creatine kinase level of 1438 UI/L (7.5×) (reference value under 190 UI/L), and serum aldolase level of 10.1 (1.3×) (reference value under 7.6 U/L).Computed tomography of the pelvis, thighs and legs revealed advanced gluteus maximus, gluteus medius, and gluteus minimus involvement. At the thigh, there was advanced (grade 4) biceps femoris, semitendinosus, and semimembranosus involvement; severe (grade 3) vastus lateralis, vastus medialis, vastus intermedius, and adductor magnus involvement, with relative preservation and hypertrophy of the sartorius and gracilis. At the leg, there was asymmetric muscle involvement with advanced (grade 4) soleus, right gastrocnemius medialis, and left peroneus involvement, as well as tibialis anterior muscle sparing (Figure 1).Patient was submitted, when he was 31 years old, to a left vastus lateralis muscle biopsy that demonstrated abnormal architecture with areas of fibrous and fat replacement, endomysial fibrosis, fiber splitting, atrophic and hypertrophic fibers. There were groups of atrophic and groups of hypertrophic fibers (Figure 2A), with type 2 fiber predominance (data not shown). Frequent rimmed vacuoles were observed. These rimmed vacuoles presented different sizes and shapes, peripheral or central locations, inside the sarcoplasm of both atrophic and hypertrophic fibers. Rimmed vacuoles were characterized by empty spaces with a rim of basophilic material (Figure 2B). No lobulated fibers were observed on the muscle biopsy sample.Immunohistochemical studies demonstrated normal reaction for sarcoglycans (alpha, beta, gamma, delta), dysferlin, merosin, caveolin, myotilin, and emerin. Immunohistochemical studies for the three dystrophin epitopes (Rod, carboxy-terminus, amino-terminus), disclosed focal irregular reaction for the carboxy-terminus that was evident on serial sections of the same fibers (Figure 2D,E,F). Immunohistochemical reaction for the anti-telethonin antibody showed total deficiency (Figure 2C), consistent with telethoninopathy (Limb Girdle Muscular Dystrophy type 2G).Transmission electron microscopy demonstrated endomysial fibrosis, fiber and myofiber splitting, focal intrasarcoplasmic glycogen deposits, many degenerating fibers, very small atrophic fibers with or without degeneration signs, and autophagic vacuoles with numerous membrane and sarcoplasmic debris (Figure 2G,H).

The molecular investigations were part of standard patient diagnostics, and included the search for Facioscapulohumeral muscular dystrophy, Spinal muscular atrophy, mitochondrial m.3243 A < G mutation, dystrophinopathy, and Limb Girdle Muscular Dystrophy type 2A (calpainopathy), which disclosed normal results. The *FKRP* gene dHPLC followed by direct sequencing to investigate the hypotheses of Limb Girdle Muscular Dystrophy type 2I (fukutin-related myopathy) revealed one single heterozygous exon 4 c.461G > T, p.(Arg154Phe) mutation in the *FKRP* gene with unknown significance. Sequencing of the 2 exons of the *TCAP* gene identified the common c.157C > T (p.Q53X) mutation in homozygous state.

## Discussion

The patient here described presented his first symptoms in childhood and evident lower limb muscular weakness at 20 years old. His symptoms progression was within the age range previously described in telethoninopathy patients, that is usually between 9 and 20 years old [12]. Differently from other LGMD2G patients, however, he presented tremor on the hands at his first examination, which significance in the myopathic context of his disease is still not clear. Small hand muscle weakness was slight or inexistent in previously described patients [2]. An additional atypical characteristic of LGMD2G, observed in this patient, was a subtle extraocular and facial weakness, since facial muscles were always spared in telethoninopathy patients already reported [2,7,8]. Interestingly, this finding had been observed in another telethoninopathy Brazilian patient, examined in advanced stage of the disease [12].

The muscular weakness pattern in telethoninopathy may be predominant proximal or distal [2-9,12]. Even in patients with predominant proximal lower limb weakness, foot drop or frequent tripping, related to tibialis anterior involvement, are common initial [8,12] or late (ten years after first symptoms) [7] findings. In accordance with these clinical observations, our patient also presented predominant proximal lower limb weakness, and calf volume increase with soleus and gastrocnemius medialis involvement. On the other hand, a pattern of early tibialis anterior involvement, compared to posterior calf muscles, on muscle image studies, which has been consistently reported in teletoninopathy patients [7,8,12] was absent in this patient.

The electromyography demonstrated mixed neurogenic and myopathic motor unit potentials. Muscle biopsy demonstrated groups of atrophic and groups of hypertrophic fibers simulating a neurogenic pattern. Neurogenic pattern associated with muscular dystrophy has already been described in calpainopathy (Limb Girdle Muscular Dystrophy type 2A - LGMD2A) patients [15,16]. This pattern could be perhaps secondary to a reinnervation process after fiber group necrosis or nerve degeneration, although neither nerve degeneration nor groups of necrotic fibers were observed on this muscle biopsy. Therefore, the exact mechanism of neurogenic motor unit potentials in this patient remains elusive.

Muscle biopsy, from the patient here reported, demonstrated the frequent findings of LGMD, including fibers with rimmed vacuoles, which we well characterized by transmission electron microscopy, as vacuoles containing sarcoplasmic degradation products.

Immunohistochemical investigation demonstrated complete deficiency of telethonin, which is totally indicative of LGMD2G [2-12].

Only a small number of known patients with telethoninopathy have been reported [2-12]. As molecular diagnosis is not worldwide available, and since a huge clinical variability can be observed in all forms of LGMDs, protein techniques with commercially available anti-telethonin antibodies should be introduced in a routine basis in the diagnostic workup of any patient with undetermined limb girdle muscular dystrophy, both with proximal and distal weakness, and/or myopathic or mixed neurogenic phenotypes.

Telethonin is a sarcomeric protein that binds to the titin amino terminus [17], it is involved with normal sarcomeric regulation and development, activated by MyoD and expressed after myogenin binding during embryogenesis [18]. Therefore, it is believed that telethonin does not interact with the dystrophin associated complex [19]. Thus, the telethoninopathy physiopathological mechanism of fiber injury may be different from the putative mechanical stress disruption of the sarcolemma, during muscle contraction, in dystrophinopathy. Perhaps fiber necrosis may be secondary to a defect in sarcomeric regulation with accumulation of fiber degradation products and possible lysosomal hyperactivity.

The patient here described was submitted to muscle biopsy about eleven years after the progression of muscle weakness. No lobulated fibers or nemaline rods were observed on his muscle biopsy, in the opposite to a patient who was submitted to muscle biopsy 46 years after her first symptoms [12]. Therefore, it is possible that lobulated fibers and nemaline rods are a secondary phenomenon related to the long duration of symptoms [13], not yet manifested in this still ambulant patient.

## Conclusions

The phenotypic variability of telethoninopathy is expanded with this report, as it disclosed that: 1) early tibialis anterior involvement compared to posterior leg muscles is not mandatory at first presentation, and 2) telethoninopathy may mimic clinical and neurophysiological signs of neurogenic disorders. Later studies will be necessary to elucidate the physiopathological mechanisms involved in the development of telethoninopathy as well as the precise mechanism of muscle involvement and sparing, detectable through clinical examination and image studies, and the discovery of mechanisms to stop muscle weakness progression.

## Consent

The study was performed in accordance with the Declaration of Helsinki guidelines. Written informed consent was obtained from the patient for molecular study, publication of this Case report and any accompanying images. A copy of the written consent is available for review by the Editor of this journal.

## Abbreviations

CK: Creatine kinase; CMP: Compound muscle potential; CT: Computed tomography; MR: Magnetic resonance imaging; *FKRP* gene: Fukutin-related protein gene; LGMD: Limb girdle muscular dystrophy; LGMDs: Limb girdle muscular dystrophies; *TCAP* gene: Telethonin gene.

## Competing interests

The authors declare that they have no competing interests.

## Authors’ contributions

MMN, RXN and SVN performed the clinical diagnosis and collecting clinical data of the patient and revised the manuscript. JV and EC have been involved in analysis and interpretation of neurophysiological studies and revised the manuscript. RIT, LUY, CFA, and MV carried out the molecular investigation, they have been involved in interpretation of molecular data and revised the manuscript. ALda-CJ have been involved in acquisition and interpretation of data regarding muscle image studies and revised the manuscript. SVB have been involved in interpretation of electron microscopy studies and revised the manuscript. AC and JFP have been involved in conduction of muscle biopsy studies, interpretation of muscle biopsy results and revised the manuscript. AC have been involved in conception, design, and drafting of the manuscript. MV has been in involved in design and has revised the manuscript critically for important intellectual content. All authors read and approved the final manuscript.

## Pre-publication history

The pre-publication history for this paper can be accessed here:

http://www.biomedcentral.com/1472-6890/14/41/prepub
